# Collaborative Multi-Expert Active Learning for Mobile Health Monitoring: Architecture, Algorithms, and Evaluation

**DOI:** 10.3390/s20071932

**Published:** 2020-03-30

**Authors:** Ramyar Saeedi, Keyvan Sasani, Assefaw H. Gebremedhin

**Affiliations:** School of Electrical Engineering and Computer Science, Washington State University, Pullman, WA 991642250, USA; ramyar.saeedi@wsu.edu (R.S.);

**Keywords:** networked wearables, cost-effective, active learning, transfer learning, medical cyber physical systems, M-health, Internet of Things, signal processing

## Abstract

Mobile health monitoring plays a central role in the future of cyber physical systems (CPS) for healthcare applications. Such monitoring systems need to process user data accurately. Unlike in other human-centered CPS, in healthcare CPS, the user functions in multiple roles all at the same time: as an operator, an actuator, the physical environment and, most importantly, the target that needs to be monitored in the process. Therefore, mobile health CPS devices face highly dynamic settings generally, and accuracy of the machine learning models the devices employ may drop dramatically every time a change in setting happens. Novel learning architecture that specifically address challenges associated with dynamic environments are therefore needed. Using *active learning* and *transfer learning* as organizing principles, we propose a *collaborative multiple-expert* architecture and accompanying algorithms for the design of machine learning models that autonomously adapt to a new configuration, context, or user need. Specifically, our architecture and its constituent algorithms are designed to manage heterogeneous knowledge sources or *experts* with varying levels of confidence and type while minimizing adaptation cost. Additionally, our framework incorporates a mechanism for *collaboration* among experts to enrich their knowledge, which in turn decreases both cost and uncertainty of data labeling in future steps. We evaluate the efficacy of the architecture using two publicly available human activity datasets. We attain activity recognition accuracy of over 85% (for the first dataset) and 92% (for the second dataset) by labeling only 15% of unlabeled data.

## 1. Introduction

Mobile health (M-health), which the World Health Organization describes as “medical and public health practice supported by mobile devices” [1], is permeating modern healthcare. By mobile devices we mean smartphones, wearables, and implantable chips that are able to collect data—whether in the form of sensor data or questionnaire data—and deliver health care and preventive care services. Recent studies show that 83% of physicians in the U.S. use M-health CPS devices to provide care for their patients [2,3,4]. More broadly, healthcare providers use M-health technology to accomplish a variety of goals: access clinical information of patients, communicate with patients, collaborate with other care providers, and perform real-time health monitoring. Conversely, patients/users use mobile devices to track their own health-data, access clinical records through online portals, and provide feedback to healthcare providers (e.g., the form of daily questionnaires) [3,5,6,7,8]. This makes mobile devices a powerful gateway for providing effective services to a variety of population groups and especially the elderly, patients with chronic conditions, and those needing constant monitoring [9,10,11,12,13,14].

This potential however is harvested to only a limited extent. Most current machine learning and decision making algorithms forming the core of M-health technology are largely limited to passive, supervised models trained in controlled environments. As a result, the accuracy of these algorithms drops, sometimes dramatically, whenever a system’s configuration (e.g., number of sensors), context (e.g., device orientation), or user need (e.g., new activity) changes.

Next generation healthcare CPS devices need to be adaptive and capable of re-configuring their machine learning model(s) to new configurations, contexts or user needs so that they can continue to operate seamlessly and accurately. Here, the term accuracy is used in a general sense. It could refer to, for example, data classification accuracy or timeliness of an alert. The conventional approach to dealing with changing environments is to re-train the machine learning algorithm(s) for the new environment. This requires collecting new labeled training data, a process that is known to be time-consuming, labor-intensive, and expensive. Furthermore, it is not always possible to collect data for every context, configuration, and user need [15,16,17,18,19].

A better and more cost-effective approach is to leverage related knowledge (e.g., training data for similar contexts) to build an acceptable machine learning model and enhance the model by acquiring context-specific data from the set of knowledge sources. Our goal in this work is to design re-configurable and interactive mobile health monitoring systems in such a way that cost—including side-effects—of system adaptation is minimized.

*Active learning* is an appealing paradigm to consider towards this goal. The key idea in active learning is to first select informative instance(s) from the set of unlabeled data for a model to label, and then re-run the learning algorithm to minimize prediction and decision making errors [20,21,22]. Existing active learning approaches, however, rely on assumptions that are unrealistic for domains such as mobile health monitoring. In real M-health CPS applications, it is possible—and likely—to have multiple sources of knowledge with different levels of confidence (certainty) or areas of expertise.

We call a source of knowledge an *expert*. This could be a human (a user, a physician) or a machine (another sensor, a smart environment). Considering uncertainty of experts, a multi-expert system that implements *collaboration* between the experts can result in lower overall data annotation cost and more accurate prediction model. Indeed, with the emergence of IoT platforms, both cooperation between sensory devices and their interaction with humans, is becoming increasingly feasible and is likely to be a crucial aspect of future M-health systems [23,24,25]. Therefore, we target a system that strives to minimize the cost of active learning from experts while maintaining the system’s accuracy. The cost associated with an expert may be defined in terms of user inconvenience, cost of the expert’s feedback, cost of equipment, power consumption, etc.

Extending our preliminary work in [26], in this paper, we develop a *cost-effective multi-expert active learning* (Co-MEAL) architecture for mobile health monitoring systems. The architecture consists of a *data processing* unit and an *expert management* unit. It is designed to meet a variety of requirements: (a) Use training data from similar contexts to gain more information for higher accuracy; (b) Manage a set of heterogeneous experts including human experts, sensory systems, and smart environments with varying properties and costs; (c) Use knowledge of different experts at the same time and allow for experts to collaborate to increase their cumulative knowledge as well as data labeling accuracy; and (d) Employ prior knowledge of the system in the case of changes in setting (e.g., new classes of data, a new sensor).

We summarize our *specific contributions* below.

- **Architecture.** We propose a novel architecture designed for multi-expert M-health systems. The key idea is to keep the system’s uncertainty below a pre-specified threshold while minimizing the overall cost of re-training the learning algorithm. The architecture also provides a closed-loop collaboration between experts.
- **Formulation.** The functionality of the architecture is formulated from a learning prospective and is divided according to the different aspects of the architecture. Firstly, the architecture uses training data from the most similar context in order to initialize the learner of the system (i.e., transfer learning). Secondly, an active learning process manages the source(s) of knowledge for each query.
- **Algorithms.** Correspondingly, two algorithms are designed to handle the two aspects of the architecture. The first algorithm is proposed to find the most relevant training samples. The second algorithm is a cost-sensitive active learning approach to minimize the overall cost.
- **Evaluation.** We evaluate the proposed architecture using two different human physical activity datasets as case studies. The first dataset is on daily activities (e.g., walking, sitting), and the second dataset is on workout activities (e.g., running on a treadmill). We show that using the architecture increases accuracy of activity recognition by up to 45% for the daily activities and by up to 35% for sport activities compared to the case where the architecture is not used. We also show that the number of queries from costly experts is reduced by 78%.

The remainder of the paper is organized as follows. We discuss background and related work in Section 2. We review and categorize the different sources of variation (change) in M-health systems in Section 3. The proposed Co-MEAL architecture is presented in detail in Section 4. In Section 5 we present our formulation of the multi-expert active learning problem as a joint optimization of which data instances to select and which experts to use to annotate the informative samples. All of the algorithms we have developed are presented in Section 6. Details of the experimental setup we employed are discussed in Section 7, and the experimental validation results are presented in Section 8. We close off in Section 9 by drawing some conclusions and pointing out avenues for future work.

## 2. Background and Related Work

We discuss in this section works related to aspects of the Co-MEAL framework. The discussion is organized around the two core elements of Co-MEAL: active learning and transfer learning. Along the way we also provide basic background on these learning paradigms.

### 2.1. Active Learning

The key idea behind active learning is to allow a learning algorithm to choose the data instances from which it learns. In doing so, the system should strive to minimize the cost of querying while preserving the system’s accuracy [27,28,29]. There are applications in which unlabeled data is plentiful but manual labeling of data instances is expensive. In such a scenario, learning algorithms can be designed to actively query the experts to label data. Longstaff et al. [30] show how an active learning approach improves accuracy of human activity recognition compared to semi-supervised methods. However, in their work samples are selected in the active learning phase without an effective query strategy.

One approach to design a query strategy is to measure information gain of instances, since informative instances add more knowledge to a machine learning model [20,31]. In [28], the authors propose a novel framework for active learning using sampling theory for graph signals. A graph is built based on the similarity of data instances and their labels are modeled as graph signals. The sample selection is designed in such a way that the entire graph signals (i.e., labels) can be reconstructed with most informative data instances.

In [32], several query strategies are used in the context of smart-homes and crowd sourcing applications. Another query strategy is used in [33] for activity recognition in single-person smart homes. This approach uses clustering to reduce the amount of labels needed; specifically, labels are needed for each cluster, rather than each data point. A limitation of all of the above active learning approaches is that they do not consider the cost of annotation for data labeling in a *multi-expert* setting.

Cost-sensitive multi-expert active learning has been investigated in the broader literature of machine learning [34,35,36], but to the best of our knowledge it has not been studied for design of M-health systems [37]. The authors in [16] present an empirical study of active learning in real world scenarios where cost of annotations are considered in the process. In another study [34], several scenarios are presented for an active learning setting where multiple oracles with different costs and capabilities are responsible for data annotation.

### 2.2. Transfer Learning

In an M-health system, a learner often begins with a small number of instances or no labeled data at all for the current context [38,39,40]. This requires advanced methods for initializing the learning algorithm.

When faced with a new situation, recognizing and applying relevant knowledge gained in a related earlier situation is clearly profitable—humans do this rather routinely. Intuitively, *transfer learning* follows a similar idea. It aims at developing methods to transfer knowledge gained in a source task to improve learning in a related target task. We refer to the training domain where labeled data is available as the *source domain*, and to the test domain where labeled data is unavailable or very little is available as the *target domain* [41].

We formally define transfer learning and a few associated notions in the remainder of this subsection.

**Definition** **1** (Task). *Given a domain, D={X,P(X)}, where X is a vector in feature space denoting the input and P(X) is the distribution of X, a task consists of two components, a label space Y and a prediction model M(X), and is expressed as T={Y,M(X)}.*

The model *M* is used to predict the label of a new instance *x* drawn from *X*.

**Definition** **2** (Transfer Learning). *Given a source domain $D_{s}$ and learning task $T_{s}$, and a target domain $D_{t}$ and learning task $T_{t}$ , transfer learning aims to improve the accuracy of the target predictive model $M_{t}$ in $D_{t}$ using the knowledge in $D_{t}$ and $D_{s}$ in the case where $D_{s}\neq D_{t}$ and/or $T_{s}\neq T_{t}$.*

In transfer learning, as noted in the studies [42,43,44], there are three main questions to answer: (1) what to transfer; (2) how to transfer; (3) when to transfer. To find answers to these questions, we need to extract application-specific metrics to measure the quality of available knowledge. Furthermore, we need to develop algorithms to transfer the knowledge in a way that increases the performance of the target domain. Finally, we need to notify the system whenever it is necessary to transfer the available knowledge

Transfer learning has in recent works been used for system reconfiguration of health monitoring systems. In [45], the authors present several heterogeneous transfer learning algorithms which map features from the target domain to the source domain for activity recognition in the context of smart-homes. In [38,39], the authors present signal-level algorithms for cross-device and cross-subject knowledge transfer between the source domain and the target domain. In another work, an approach that integrates new sensory devices to a system without the need for new labeled training data is presented [46]. Zheng et al. [19] developed a learning method to find a Web-based similarity function to map activity data of source domain to that of target domain. A confidence level is used to measure the closeness of two domains.

Although knowledge transfer algorithms provide advantages, it is inadequate to develop machine learning algorithms based solely on similarity of two different domains. The Co-MEAL architecture is designed to use both transfer and active learning. We have proposed our own algorithms for both; however, depending on the variation situation any algorithm in the literature can be used as part of the framework.

## 3. Scenarios Driving Reconfiguration

**Figure 1:**
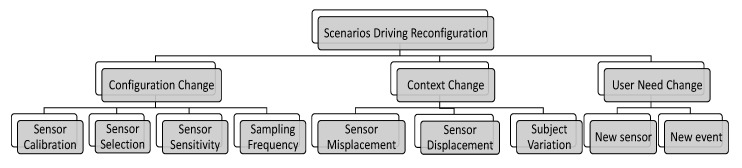
Classification of heterogeneity (variations) in M-health monitoring systems.

Before presenting the details of the Co-MEAL framework, we review in this section the different causes of *data variation* (heterogeneity) in M-health systems. We depict in Figure 1 a hierarchical categorization of the variations. At a high level, the variations can be grouped in three classes: configuration change, context change, and user need change.

### 3.1. Configuration Change

Wearable devices are often built using low cost sensors, which are inaccurate and poorly calibrated. As a result, the signal readings are noisy and with low sensitivity. On the other hand, activity recognition models are built based on training data collected on commercial platforms, which consist of accurate and dedicated standalone sensors with higher sampling frequency. Furthermore, it is possible to consider a scenario where sensors leave or join the M-health system. We call this device-related heterogeneity *configuration change*. Depending on the complexity of the situation, both autonomous and interactive reconfiguration can be applied to deal with this type of heterogeneity. In this work we focus on the variant of configuration changes resulting from addition of sensory devices.

### 3.2. Context Change

Sensor data generated during personalized exercises or tasks performed by one subject (or patient) may be too specific or inadequate to be used as a training set for a new subject. Even for the same subject, physiological patterns may change over time. In another related situation, subjects may install their mobile devices in different locations or orientations, which leads to inaccurate classification. Furthermore, a subject’s life style may change over time. We classify all of these types of variation under *context change*. Among the various variants in this category, in this work, we focus on inter-subject variation.

### 3.3. User Need Change

With the emergence of IoT platforms, M-health monitoring systems need to adapt whenever a user’s preferences change. For example, a user may need to enhance the precision of the system as they change their life style. Furthermore, a user may need to increase the capability of the system. For example, a user may need to classify new set of activities due to changes in their workout program. We refer to any user-initiated change as *user need change*. In this paper, we consider the case where a user adds more classes of data to classify by the M-health system. Due to the complexity of this situation, we almost always need to perform interactive reconfiguration unless the training labeled data already includes related databases for autonomous reconfiguration. In this paper, we assume that there is no related data for the new classes.

## 4. The Co-Meal Architecture

A conventional mobile monitoring system is generally composed of several sensor nodes, a base station, and a back-end server. Each sensor node is attached to the body (a wearable or an implant device) to sample and preprocess physiological signals and transmit partial results (e.g., features) to the gateway. Moreover, sometimes additional sensors are placed in the environment (e.g., smarthome) to capture the context as well as the decision making process. The gateway is a more powerful unit, such as a smartphone, that performs data fusion for the health analytics system. The results are further transmitted, typically through the Internet, to a back-end server for storage, further processing, and clinical decision support from physicians and health-care experts.

**Figure 2:**
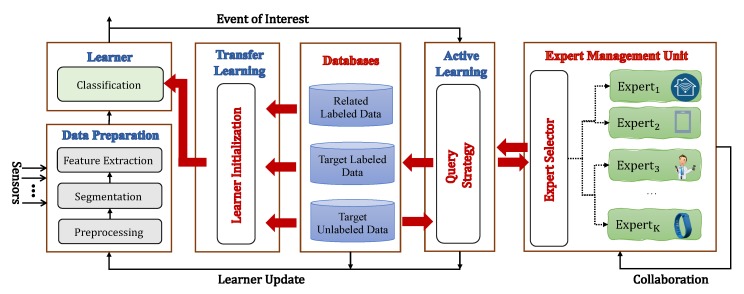
High-level representation of the proposed collaborative multi-expert active learning (Co-MEAL) architecture for mobile health monitoring.

With this description of conventional architecture of M-health systems as a background, our proposed reconfigurable architecture is constituted as depicted in Figure 2. It consists of two main components: (1) a data processing unit (DPU), and (2) an expert management unit (EMU). The DPU is the core intelligence of the M-health system. The DPU may be located entirely on the gateway or on both the gateway and the back-end server depending on the computational power of the gateway. The EMU and the experts can be considered part of the back-end server.

The DPU communicates with databases (DB) storing labeled and unlabeled data and the EMU whenever there is a need for system reconfiguration. The incoming data is pre-processed by the Learner in the DPU, and then the Query Strategy, also in the DPU, decides whether or not to request labels. If the Learner is capable of classifying the data accurately using the labeled data in the current context, the active learning step is deactivated. We discuss in the remainder of this section details of the Learner Initialization, the Query Strategy, the Databases, and the EMU.

### 4.1. Learner Initialization

In active learning, a learner often begins with a small number of instances in the labeled training set *L*. However, an M-health system quite often may have to start with no labeled data at all. This requires more sophisticated strategies in the initialization phase of the active learning process. Meanwhile, for many applications of M-health, there may be data for *related* contexts. For example, in a physical activity monitoring system, the data from other subjects could be used to initialize the classifier of the system. In Section 6, we present an initialization algorithm which uses the related labeled data to build the first learner model.

### 4.2. Query Strategy

To reduce annotation cost, we need a query strategy to select the best subset of unlabeled instances. The decision of whether or not to query an instance can be framed in several ways. One approach is to evaluate samples using some measure that shows how informative an instance is, since more informative instances are more likely to be queried [20]. For such a purpose, an entropy-based measure can be used. Entropy is an information-theoretic measure that represents the amount of information needed to encode a distribution. An informative data instance is defined as follows [20].

**Definition** **3** (Informative Instance). Let x be a vector that stands for an unlabeled data sample and $P_{M}\left(\ell_{i}|x\right)$ be the posterior probability under prediction model M. Then the data point $x_{H}^{*}$ expressed below is the most informative data point:

(1) $$x_{H}^{*}=\underset{x}{\mathrm{argmin}}\sum_{i=1}^{m}P_{M}\left(\ell_{i}|x\right)\log P_{M}\left(\ell_{i}|x\right)$$

*where $\ell_{i}$ ranges over all possible labels.*

Another query strategy is a committee-based algorithm. This approach involves maintaining a committee of models (e.g., classifiers) which are all trained on the current labeled set *L*, but represent competing hypotheses. Each committee member is then allowed to vote on the labels of query candidates. The most informative instance is considered to be the one about which they most disagree. Different models should represent different regions of the feature space, and have some measure of disagreement among committee members [20].

In this paper, we use a hybrid query strategy of the above approaches that is based on a *random forest* classifier. The informativeness of an input instance is computed as the mean informativeness of the trees in the forest. The informativeness measure of a single tree is the fraction of samples of the same class in a leaf.

### 4.3. The Databases (Dictionaries)

A key component in our active learning architecture is a set of databases (aka dictionaries in the time-series literature). Each database represents knowledge for a unique case. Below, we formally define a database.

**Definition** **4** (Database). *A database (or dictionary) DB is an unordered set of instances. The $i^{th}$ instance is a q-dimensional vector denoted by $DB_{i}$, where q is number of features extracted from each instance. Each instance may or may not be labeled in the dictionary.*

In our architecture, there are three types of dictionaries. The *target unlabeled data* dictionary stores the incoming streaming data in real time. This data will be used both to initialize the system and to modify the classifier. The second database, *target labeled data*, gradually collects two kinds of information: (i) instances from the third database, *related labeled data*, and (ii) queries from the EMU. The database referred to as related labeled data is built based on related contexts and domains useful for initializing the classifier and decreasing the cost of queries.

### 4.4. The Expert Management Unit

In real-world health-care applications, labels may come from various sources, including different health-care experts, other mobile devices or even a smart environment. Furthermore, the knowledge provided by each expert is subject to uncertainty. For example, the performance of human annotators depends on factors such as level of expertise, experience, and concentration/distraction [47]. Additionally, experts may have more or less knowledge for different parts of the problem that has to be solved (e.g., different classes, different features). Another factor is the cost of data annotation by each expert. Therefore, it is necessary to design a system that is capable of interacting with multiple experts efficiently.

The multi-expert architecture we propose satisfies several unique properties.

- The learning process relies on the collective intelligence of a group of experts.
- The architecture could also provide a learning opportunity for experts. In particular, our system allows for each expert to update their knowledge based on the feedback from other experts in each round of query.
- The expert selection submodule chooses the expert(s) in such a way that the query cost is minimized, while keeping the provided label for the queried instance accurate.
- The architecture leverages different points of view from the experts to provide a more comprehensive model through the system’s life time. For example, new class labels may arise during the life time, or a new sensor may be integrated into the mobile health system.
- An expert can be part of the data processing unit. For example, in a multi-node mobile system, one node shares the label with the DPU whenever the node is confident.

We present the algorithm we developed for expert selection in Section 6.

## 5. Problem Statement

Let *L* = $\left\{\ell_{1},\ell_{2},\ldots ,\ell_{k}\right\}$ be *k* groups of data that the system needs to classify (label). Let *E* = $\left\{e_{1},e_{2},\cdots ,e_{p}\right\}$ be a set of *p* possible experts each of which has full or partial knowledge of the current labeled set *L*. We model the knowledge quality of each expert on the label set *L* using an *uncertainty score vector*. For a given expert, its uncertainty score vector $\vec{U}=\left(u_{1},u_{2},\ldots ,u_{k}\right)$ is a vector whose $i^{th}$ entry stores a value in the range [0,100] (i.e., percentage) indicating the uncertainty of the expert on its prediction of the label $\ell_{i}$. A number close to 0 indicates high expertise. The Learner in our architecture is considered to be one of the experts in the sense that it has its own uncertainty score vector.

We are now ready to formally define the collaborative multi-expert active learning problem:

**Problem** **1** (Co-MEAL Problem). *Let $X_{U}=\left\{x_{1},x_{2},\cdots ,x_{n}\right\}$ be the sequence of data instances in the new context or configuration, the labels of which are not yet known with certainty. Let $X_{L}=\left\{\left(x_{n+1},y_{n+1}\right),\left(x_{n+2},y_{n+2}\right),\cdots ,\left(x_{n+m},y_{n+m}\right)\right\}$ be the set of labeled data. This labeled data may come from related labeled data $X_{R}$ or partially trained learner (i.e., prior knowledge). The Co-MEAL problem is to select a subset $X_{S}\subset X_{U}$ to ask from the set of heterogeneous experts E such that the overall data annotation cost is minimized and uncertainty of the learner model $M_{lr}$ trained on the set $X_{S}\cup X_{L}$ is lower than a pre-specified threshold in the new context.*

We formulate the problem as a joint optimization of which data instance(s) to select and which expert to use to annotate. The objective is to minimize the active learning cost under the pre-defined uncertainty threshold:

(2) $$\mathrm{Minimize}\;\;C_{al}\left(M_{lr}\right)\;\;\mathrm{subject}\;\mathrm{to}\;\;{\vec{U}}_{M_{lr}}\leq {\vec{U}}_{th}$$

where ${\vec{U}}_{th}$ is the acceptable (threshold) amount of uncertainty in the system’s performance, $M_{lr}$ is the learner model, ${\vec{U}}_{M_{lr}}$ is the uncertainty of the learner, and $C_{al}\left(M_{lr}\right)$ is the cost of query of the selected instances from the set of experts.

Equation (2) can be rewritten by incorporating query strategy and expert selection into the objective function. Let $\beta_{i}$ be a binary variable that determines whether or not sample $x_{i}$ is selected as an informative instance:

(3) $$\begin{array}{ccc} \beta_{i} & = & \left\{\begin{array}{cc} 1, & \mathrm{if}\;\mathrm{label}\;\mathrm{for}\;x_{i}\;\mathrm{is}\;\mathrm{needed}\\ 0, & \mathrm{otherwise}. \end{array}\right. \end{array}$$

Let $\alpha_{ij}$ be a binary variable indicating whether or not the expert selection model requests the label for instance $x_{i}$ from expert $e_{j}$. That is:

(4) $$\begin{array}{ccc} \alpha_{ij} & = & \left\{\begin{array}{cc} 1, & \mathrm{if}\;\mathrm{label}\;\mathrm{for}\;x_{i}\;\mathrm{is}\;\mathrm{asked}\;\mathrm{from}\;e_{j}\\ 0, & \mathrm{otherwise} \end{array}\right. \end{array}$$

And let $c_{ij}$ be the query cost for instance $x_{i}$ from expert $e_{j}$ and *p* be the number of experts. Then, the Integer Linear Programming (ILP) formulation for the Co-MEAL Problem is as follows:

(5) $$\mathrm{Minimize}\;\;\;\sum_{i=1}^{n}\sum_{j=1}^{p}\beta_{i}\alpha_{ij}c_{ij}$$

subject to:

(6) $$\sum_{j=1}^{p}\alpha_{ij}=1\;\;\;\;\;\;\forall \;i\;\in \;\left\{i\;|\;\beta_{i}=1\right\}$$

(7) $$\beta_{i}\in \left\{0,1\right\},\;\;\alpha_{ij}\in \left\{0,1\right\}$$

(8) $${\vec{U}}_{M_{lr}}\leq {\vec{U}}_{th}$$

The constraint (6) guarantees that each selected unlabeled data is asked exactly from one expert and the constraint in (7) ensures that the variables $\beta_{i}$ and $\alpha_{ij}$ take only binary values.

It is extremely difficult to optimize the Co-MEAL Problem directly, since even the simplified version of the problem is NP-hard. The simplified problem is the case where the selected samples from the related data are known and we only need to find the optimal set of experts to annotate the unlabeled selected instances. The minimization is over the entire set of potential sampling sequences and experts with different costs, an exponentially large number. Furthermore, the learner $M_{lr}$ is updated with each additional example and we can only calculate this effect after we know which examples are chosen and labeled, which makes it impossible to directly solve the problem.

## 6. Algorithms

We instead developed greedy algorithms to find effective, approximate solution for the Co-MEAL Problem. We use available query strategies to find the most informative data instances as we discussed in Section 4.2. In this section, we present the algorithms we developed for learner initialization and expert selection as well as the driver algorithm for the overall functionality of the architecture.

### 6.1. Driver Algorithm: Collaborative Active Learning

The driver routine for solving the Co-MEAL problem is presented in Algorithm 1. The algorithm takes as input the dictionary containing other contexts’ data $DB_{R}$, the dictionary of target labeled data $X_{L}$ (which could be an empty set or could contain a small number of instances at first), the dictionary of target unlabeled data $X_{U}$, the label set *L*, the number of instances for each round of query *K*, the uncertainty threshold vector ${\vec{U}}_{th}$, and the expert set *E*. It delivers as output the model $M_{lr}$ which is retrained with the augmented labeled data.

Algorithm 1 calls the routine INITITIALIZELEARNER (Algorithm 2) to determine the closest data instances in the related contexts $DB_{R}$. It then expands the target labeled database $X_{L}$ with them. In the next step, the architecture uses the current learner model to label the target domain whenever prior knowledge is applicable (lines 2–6). This is useful whenever the data distribution is the same but we need to upgrade the system’s configuration (e.g., new sensors, new set of labeled data).
**Algorithm 1** COLLABORATIVEACTIVELEARNING-DRIVER**Input:**$DB_{R}$, $X_{L}$, $X_{U}$, $L=\{\ell_{1},\ell_{2},\cdots ,\ell_{k}\}$, *K*, ${\vec{U}}_{th}$, *E* **Output:** LEARNER
$M_{lr}$
1:$DB_{L}\leftarrow I\mathrm{NITITIALIZE}L\mathrm{EARNER}\left(X_{U},DB_{R},L\right)$2:**for all**$x_{i}\in X_{U}$**do**3: **if** old knowledge is applicable **then**4:  $X_{L}\leftarrow X_{L}\cup \left(x_{i},y_{i}\right)$, $X_{U}\leftarrow X_{U}\backslash \left(x_{i},y_{i}\right)$5: **end if**
6:**end for**7:$X_{L}\leftarrow X_{L}\cup DB_{L}$, $X_{S}\leftarrow Ø$8:$M_{lr}\leftarrow C\mathrm{LASSIFIER}\left(X_{L}\right)$9:**while**${\vec{U}}_{M_{lr}}>{\vec{U}}_{th}$**do**10: $X\leftarrow \mathrm{GET}I\mathrm{NFORMATIVE}I\mathrm{NSTANCES}(K,X_{U})$
11: **for all** $x_{i}\in X$ **do**
12:  $y_{i}\leftarrow A\mathrm{NNOTATE}\left(x_{i},E,M_{lr}\left(x_{i}\right),{\vec{U}}_{th}\right)$
13:  $X_{S}\leftarrow X_{S}\cup \left(x_{i},y_{i}\right)$
14: **end for**
15: $\mathrm{BROADCAST}L\mathrm{ABELED}D\mathrm{ATA}(X_{S})$    /*collaborative*/16: $\mathrm{UPDATE}U\mathrm{NCERTAINTY}A\mathrm{LL}(X_{S},E)$    /*collaborative*/17: $X_{L}\leftarrow X_{L}\cup X_{S}$, $X_{U}\leftarrow X_{U}\backslash X_{S}$, $X_{S}\leftarrow Ø$18: $M_{lr}\leftarrow U\mathrm{PDATE}L\mathrm{EARNER}\left(X_{L}\right)$
19:**end while**


The while-loop represents the data annotation phase. There are two steps in each iteration of the while-loop: (1) the most informative instances are chosen via the function GETIFORMATIVEINSTANCES (see the query strategy submodule in Section 4.2), which returns the *K* most informative instances from the database of target unlabeled data; and (2) the routine ANNOTATE (Algorithm 3) obtains labels from confident-enough, cost-efficient experts for each selected instance.

Then, the new labeled data instances augment the target labeled database and re-train the learner model $M_{lr}$. The routine BROADCASTLABELEDDATA conveys the new labeled data to the other experts. The UPDATEUNCERTAINTYALL routine is run on each expert to re-calculate their uncertainty score based on the new gathered informations (i.e., new labeled instances). This process is repeated until the learner’s uncertainty scores falls below the uncertainty threshold $U_{th}$ for all $\ell_{i}\in L$. The learner re-trained based on the enhanced target labeled data database is returned as the output of the driver algorithm for the Co-MEAL architecture.

### 6.2. Learner Initialization: Transfer Learning

As was mentioned earlier, we need to use the available knowledge when data distribution in the source domain(s) is different from that in the target domain (i.e., current context). The INNOTATELEARNER routine (Algorithm 2) takes as input the database of labeled data for related contexts $DB_{R}$, the database of unlabeled data for the current context $X_{U}$, and the label set *L* including *k* types of labels. The database of related contexts $DB_{R}$ may include none to several source domains (e.g., different sensor locations, different subjects). The algorithm finds *k* clusters for the unlabeled data (line 2). It then searches the entire database of related data $DB_{R}$ to find the closest set of labeled data to each cluster, and returns these data as the output. Please note that labeled data for each $\ell_{i}\in L$ may come from different source domains. We employed cosine similarity to measure the closeness of clusters in the current context and labeled data in the new context [19].
**Algorithm 2** INITITIALIZELEARNER**Input:**$DB_{R}$, $X_{U}$, $L=\{\ell_{1},\ell_{2},\cdots ,\ell_{k}\}$ **Output:**$DB_{L}$1:$DB_{L}\leftarrow Ø,SIM\leftarrow Ø$2:$\left(Centers_{u},Clusters_{u}\right)\leftarrow C\mathrm{LUSTERING}\left(X_{U},k\right)$3:**for all**${DB_{R}}_{i}\in DB_{R}$**do**4: $SIM_{i}\leftarrow cosSim\left(Centers_{{DB_{R}}_{i}},Centers_{u}\right)$
5: $SIM\leftarrow SIM\cup SIM_{i}$
6:**end for**7:**for all**$\ell_{i}\in L$**do**8: $DB_{\ell_{i}}\leftarrow argmax_{\ell_{i}}SIM$
9: $DB_{L}\leftarrow DB_{L}\cup DB_{\ell_{i}}$
10:**end for**

### 6.3. Expert Management: Active Learning

One focus of this paper is the heterogeneity (e.g., uncertainty, type, and cost) of experts. Our system should be cost-sensitive and we need to minimize the cost of active learning. In real-world scenarios for mobile health monitoring, the system can actively communicate with other sensory systems, the user (through the interface), the physician (through the back-end server), etc. In addition to type of an expert, one other major problem in health monitoring is the subjectivity of the expert’s decisions. For example, the performance of human annotators depends on factors such as level of expertise, experience, and concentration/distraction [47].

**Figure 3:**
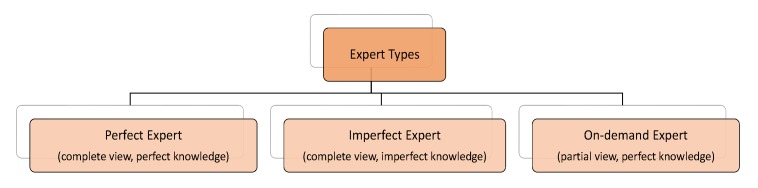
Different types of experts in a multi-expert setting in an M-health system.

The first task in our expert management in active learning context is to categorize different types of experts and then find a cost-sensitive solution for the problem. We categorize the experts into three different types as follows (see also Figure 3).

- **Perfect Expert:** A perfect expert supplies correct labels for every query, but the cost of data labeling is high. Our goal is to minimize the number of queries from such experts. An experienced physician, a multi-sensor smart health facility center, a supervised data collection system are all examples of perfect experts.
- **Imperfect Expert:** An imperfect expert provides labels for each query, but the validity of a label is questionable. In other words, the expert is knowledgeable in only part of the data distribution and it may provide incorrect labels. Examples of imperfect experts are inexperienced doctors, the user, and sensory systems with limited capabilities (e.g., limited number of sensors). The cost of labeling by an imperfect expert is lower, but still we need to minimize the number of queries from such experts.
- **On-demand Expert:** An on-demand expert, in contrast to the other types of experts, provides labels with little or zero cost. However, their knowledge is limited (e.g., a subset of label set *L*) and they may not be able to reply to every query. Other wearable sensors with different configurations are examples of on-demand experts.

The system should be capable of approximating the uncertainty of experts over the label set *L*. This is possible because the reputation and/or performance of experts can be estimated based on available reviews. For example a wristband activity tracker may be known to be an expert on detection of walking with different paces, or an experienced doctor may be well-known for her/his expertise. Based on the above categorization, we propose a greedy algorithm for expert selection that minimizes the cost of data annotation (Algorithm 3).
**Algorithm 3** ANNOTATE**Input:***x*, *E*, semi-label L, ${\vec{U}}_{th}$ **Output:** label *ℓ*
1:u←1002:**for all** On-demand expert $e_{i}\in E$ **do**3: L←MAJORITYVOTE(x)
4: u←$M\mathrm{IN}U\mathrm{NCERTAINTY}(e_{i},L)$ in labeling *x*5:**end for**6:**while**$u>U_{th}\left(L\right)$**do**7: $E_{conf}\leftarrow Ø$, $C_{conf}\leftarrow Ø$8: **for all** perfect or imperfect expert $e_{i}\in E$ **do**9:  **if** $U_{i}\left(L\right)\leq u_{th}$ **then**10:   add $e_{i}$ to the $E_{conf}$ /*confident experts*/11:   add $cost(e_{i})$ to $C_{conf}$ /*cost of experts*/12:  **end if**13: **end for**
14: expert ← $argmin_{e_{i}\in E_{conf}}C_{conf}$15: L← label from expert for instance *x*16: u← uncertainty of expert in labeling *x*17:**end while**18:ℓ←L


The algorithm ANNOTATE takes as input the instance *x*, the set of experts *E*, a semi-label L, and the uncertainty threshold ${\vec{U}}_{th}$. The semi-label L is the most probable label(s) for instance *x* based on the learner’s current knowledge. The goal of the algorithm is to find the least costly and most confident expert for the instance *x*. The expert selection method is performed in two phases. First, the expert management unit asks the on-demand experts to provide labels. The semi-label L and its related uncertainty change if on-demand experts are more confident about another specific label based on majority voting. If the uncertainty for the new semi-label L is still below the threshold, the second phase is performed to ask from the other experts (i.e., imperfect and perfect experts).

The set $E_{conf}$ is the set of experts that are confident enough to detect the semi-label L at a given moment during the course of the algorithm. The semi-label L is updated in the course of the algorithm, and is deemed acceptable if the uncertainty score of the selected expert is lower than the threshold ${\vec{U}}_{th}\left(L\right)$. Further details are shown in Algorithm 3.

### 6.4. Variants on the Driver Algorithm

Before concluding the current section on algorithms, we include a brief discussion on a matter that is related to our experiments. Specifically, in our experiments, we consider three different *variants of Algorithm 1* in order to show the effects of different properties:

- CAL corresponds to the “normal” operation of the Co-MEAL architecture with both transfer learning and collaboration, but with no prior knowledge available. This is a variant of Algorithm 1 in which lines 2–6 are excluded.
- CAL* corresponds to the case where learner(s) have been in the system with less knowledge and the active learning phase is used to upgrade the system with changes such as adding a new sensor or new labels. CAL* is variant of Algorithm 1 in which the lines 2–6 are included.
- NCAL corresponds to the operation of the Co-MEAL architecture with transfer learning and without collaboration. NCAL is simply a variant of Algorithm 1 in which the lines 15 and 16 corresponding to collaboration between experts are excluded.

The way data is collected for CAL* requires a bit of clarification. Whenever the subject uses the system with the new configuration, those samples for which the system is confident to label are added to the target labeled database ($X_{L}$).

## 7. Experimental Setting

Human activity recognition (HAR) is an important component of M-health systems. For example, it enables novel context-aware monitoring in elderly care and health care for patients with chronic diseases. It also provides standalone applications, for example, for measuring energy expenditure. Various types of sensors are used in mobile devices, including gyroscopes, magnetometers, accelerometers, GPS sensors, or combinations of different sensor modalities [48,49,50,51]. We therefore chose activity recognition as our case study for evaluation. In this section, we describe the two datasets we use in our study and the design setting we employ in all scenarios mentioned in Section 3. For all our experiments, we used the *scikit-learn* machine learning library.

### 7.1. Datasets

We consider two datasets with two distinct focuses.

**Dataset 1.** This dataset concerns daily activities and is publicly available at the Web Research Group at University of Mannheim [50]. Each subject is asked to perform each activity for approximately ten minutes. The dataset comprises outputs of accelerometer, GPS, gyroscope, light, and magnetometer for 15 subjects (7 females and 8 males, age 31.9±12.4, height 173.1±6.9, weight 74.1±13.8). The data is collected in a semi-controlled condition and subjects were in-charge of installing the sensors. The size of this dataset is 3.5Gb. The data is collected from different body locations: chest, forearm, thigh, upper arm, head, shin, and waist. We only used the accelerometer and gyroscope data collected from all locations. Each sensor collects data with sampling frequency of 50Hz. The diversity of subjects, number of sensors, and the uncontrolled nature of data collection makes this dataset a perfect choice to validate Co-MEAL architecture.

**Dataset 2.** The second dataset is the “Daily and Sports Activities Dataset” [52] which is publicly available at University of California-Irvine’s data repository. Here, subjects were asked to perform the activity set while sensors are placed on five different body locations. Eight subjects were asked to perform nine sport activities (e.g., walking on treadmill, rowing, basketball playing), each for a duration of five minutes. Each device collects data with sampling frequency of 25Hz from tri-axial inertial sensors (i.e., accelerometer, magnetometer, gyroscope). The subjects are asked to perform the activities in an uncontrolled way and were not restricted on how the activities should be performed. For this reason, there are inter-subject variations in the speeds and amplitudes of some activities which makes it a good dataset to check the validity of active/transfer learning algorithms. The dataset includes 60 repetition for each activity from each subject which covers many possible way of performing an activity.

### 7.2. Data Processing

A moving average filter is applied on the activity data to remove high frequency noises from inertial sensors (*preprocessing*). For the first dataset, after the data filtering, a two-second sliding window was applied for data *segmentation*, and we consider 50% overlap between consecutive windows. The second dataset is segmented into windows of five seconds. A variety of features can be extracted from each segment of data (*feature extraction*). We extracted statistical features that have been found in previous studies to be effective for activity recognition [53,54]. More details on the statistical feature set used in the experiments is presented in [26].

The next step in the data processing is *classification*. Previous research in the field of activity recognition has shown that Random Forest (RF), K-Nearest Neighbors, and Support Vector Machine classifiers can be effective [51,55]. In this paper, we used an RF classifier for both the learner model and the sensor-based experts. We chose RF classifier for two reasons. First, RF works as a committee of decision tree classifiers on different sub-samples of the data and uses averaging to refine the predictive accuracy to control over-fitting. Secondly, using RF, we are able to measure the uncertainty of machine learning models over the unlabeled target data (Section 4.2). We used the k-means clustering algorithm to categorize the unlabeled target data ($C\mathrm{LUSTERING}(X_{U},k)$ in Algorithm 2). We set the number of estimators for each RF classifier to 10. Otherwise, we used the default settings in *scikit learn* for both the RF and the k-means algorithms we used.

### 7.3. Setting of Experts

As we described in Section 6.3, our architecture involves three different types of experts. Each expert has its own properties such as type, uncertainty, and cost. The cost of an expert is proportional to its confidence level over the label set *L*. We split the available labeled data in both datasets into *expert initialization* and *evaluation* subsets, with a ratio of 50% for each subset for each location (e.g., head, shin). We use the *expert initialization* to train the experts and the evaluation data to validate the proposed architecture. We assume that all of the devices are experts, except for the one which we assume is the learner. The various expert types are simulated as follows.

**Perfect Expert.** We used the ground truth label as the least uncertain (most confident) expert in the expert management unit. In a real-world scenario, this expert can be a highly confident physician or the user in the monitoring system. To be more realistic, we also allow for the possibility of error made by a perfect expert. We set the probability of error between 1% to 10% depending on the difficulty of detecting the specific label.
**Imperfect Expert.** We model an imperfect expert using the sensors from other locations (as opposed to the learner’s location). However, in order to generate imperfect experts, we partly use the expert initialization data. For example, we only use 5% of the expert initialization data for each label. If we need to decrease the uncertainty of an imperfect expert, we add more labeled data to build its classifier. We estimate the uncertainty score vector for each expert based on their prediction error on the evaluation data. We assume that, in real world scenarios, an expert is aware of its own knowledge.
**On-demand Expert.** Each on-demand expert is trained in such a way that it is only confident over a subset of the label set $L_{selected}\in L$. To simulate an on-demand expert, we use all the expert initialization data for $\ell_{i}\in L_{selected}$ to train the classifier, and a few instances for other labels.

## 8. Experimental Evaluation

We implemented all of the algorithms we developed and the various simulations of the proposed architecture in Python. In this section, we present experimental results. The presentation is organized in two parts. Section 8.1 presents results demonstrating the benefits of using active learning on physical activity monitoring, whereas the focus of Section 8.2 is on cost evaluation of the architecture.

### 8.1. Performance Evaluation Scenarios

To assess the performance of the Co-MEAL architecture, we consider three change scenarios (corresponding to the cases discussed in Section 3): context change (new subject), configuration change (new sensor) and user need change (new activity). We compared the accuracy with the upper bound of accuracy in all these scenarios. The upper bound accuracy is defined as the accuracy we can get from a model trained with data specifically collected for the situation.

#### 8.1.1. Context Change (New Subject)

In this scenario, a wearable sensor is placed on human body as a learner (e.g., wristband activity tracker). We assume that we have labeled data for other subjects, however, the current subject’s activity patterns might be different compared to them. Therefore, the operating context of the system is different from what we have in the training labeled data. Our goal is to teach the sensor for the new context (i.e., the new subject).

We initialize the learner classification algorithm using Algorithm 2 for the current context. Then, the query strategy submodule finds the most informative set of instances to ask from the expert management unit.

The query strategy submodule selects the top K=10 informative instances in each round of query. We compare the activity recognition accuracy of the collaborative active learning (CAL) algorithm with two baseline algorithms: (1) an algorithm that randomly selects samples in the query procedure instead of informative samples (RAL) and (2) an algorithm that employs a simple transfer learning-based learner (STL) which adds the most similar training data of other subjects in each iteration. We repeated the above scenario for 8 subjects in the two datasets for all three algorithms. In each re-run of this scenario the excluded subject is the new context and the rest are considered to be related contexts.

**Figure 4:**
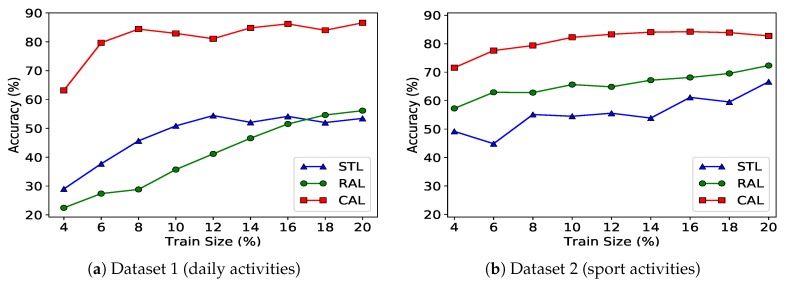
Comparison between the collaborative active learning algorithm (CAL) and two baseline algorithms: an algorithm that randomly selects samples in active learning (RAL) and a simple transfer learning-based approach (STL). The scenario is context change. Subfigure (**a**) shows results for the daily activities dataset, and subfigure (**b**) shows results for the sport activities dataset.

The activity recognition accuracy results we obtained for this scenario are summarized in Figure 4. The reported results are averages of the various re-runs. We consider the case where the sensor is located on the waist of user. The left plot in Figure 4 shows results for dataset 1 (daily activities) and the right plot shows results for the dataset 2 (sport activities). The horizontal axis shows the percentage of queries for the CAL/RAL algorithms and the percentage of used training data for the STL algorithm.

Figure 4 shows that by querying only 15% of data, the accuracy of CAL reaches 88% and 90% for dataset 1 and dataset 2. The RAL algorithm’s accuracy increases monotonously through the active learning process until a point where the added labeled data has no impact on the learner’s accuracy. This shows that the new labeled data offers no information gain if we do not have an appropriate query strategy. The accuracy of the STL algorithm varies for most cases as we increase the amount of training data from other subjects. After a point, the difference in distribution of training data and our context result in an uncertain learner for the STL algorithm.

#### 8.1.2. Configuration Change (Sensor Addition)

Here we consider a configuration change scenario in which new sensors are added to the system for better performance. To simulate the scenario, we looked at a learner with two sensors located on different body parts. Each node has a prior knowledge over a subset of label set *L* with high confidence. We assume that we have knowledge for all of the label set *L*.

**Figure 5:**
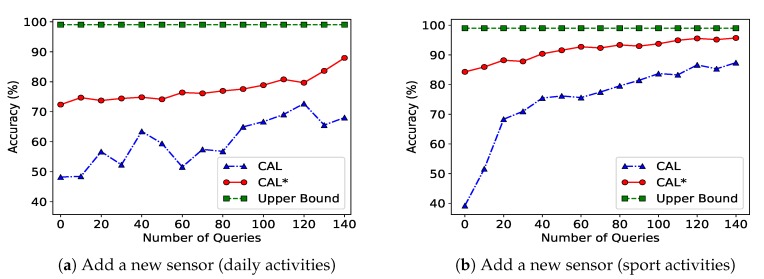
Performance of the collaborative active learning algorithm under a configuration change (adding a new sensor) scenario. Subfigure (**a**) shows results for the daily activities dataset, and subfigure (**b**) shows results for the sport activities dataset.

Recalling the descriptions of CAL* and CAL given in Section 6.4, we compare the activity recognition accuracy for (1) CAL, (2) CAL*, and (3) using training data for the same configuration, a case that can be considered to be upper bound on the accuracy (Upper Bound). We compare these three algorithms for the scenario just described. Figure 5a,b show that adding a new sensor with prior knowledge over the label set *L* increases the activity recognition accuracy by up to 25% for the daily activities and by up to 48% for the sport activities.

#### 8.1.3. User Need Change (New Activity)

**Figure 6:**
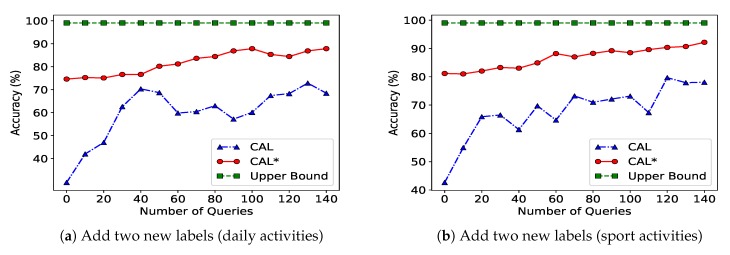
Performance of the collaborative active learning algorithm under a user need change (adding new labels) scenario. Subfigure (**a**) shows results for the daily activities dataset, and subfigure (**b**) shows results for the sport activities dataset.

The scenario considered here is a user need change in which a user adds new activity (new data to classify). To simulate this, we again look at a learner with two sensors located on different body parts (as in the previous paragraph), but in this case we let the sensory system require to label two new groups of data (i.e., activity type). Figure 6a,b show that for this case in which the sensors needed to learn new classes of data, the initial accuracy of CAL* increases by up to 40% compared to CAL for both datasets.

### 8.2. Query Cost Evaluation

In this section, we present results assessing the effects of uncertainty threshold for imperfect experts, number of on-demand experts, and benefits of collaboration on cost of active learning in the Co-MEAL architecture.

#### 8.2.1. Uncertainty Threshold, Imperfect Experts

**Figure 7:**
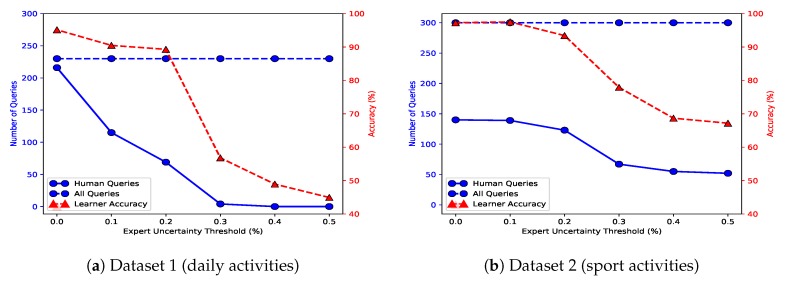
Expert uncertainty threshold versus activity recognition accuracy (right axis) and number of queries (left axis). Subfigure (**a**) shows results for the daily activities dataset, and subfigure (**b**) shows results for the sport activities dataset.

Depending on importance of accurate data classification in our system, we can control the cost of queries from perfect and imperfect experts in the EMU unit. In other-words, we can trust experts with higher level of uncertainty in the data annotation process because of the higher uncertainty threshold that the system can tolerate. We run the EMU unit for a range of uncertainty thresholds. We only consider perfect and imperfect experts to remove the role of on-demand experts from the overall cost of data annotation. Figure 7 presents the effects of uncertainty threshold on (i) the number of queries from human annotators and (ii) the activity recognition accuracy. Please note that the plots in Figure 7 use two vertical axes to show the two quantities simultaneously.

In these experiments, we assumed that the most expensive data annotator is a human, and see how our expert selection can shift the queries from the human expert to imperfect experts if we alter the uncertainty threshold. The lower the uncertainty score threshold is, the higher the number of queries from human(s). However, even with higher uncertainty threshold, we still reach an acceptable activity recognition accuracy. We have reported results for both datasets. We only employ 20% of data to annotate in both datasets. For ${\vec{U}}_{th}=0.2$, the accuracy of activity recognition increases by up to 90% for the daily activities and by up to 95% for sport activities. The number of queries from human(s) reduces by up to 32%.

#### 8.2.2. Number of On-Demand Experts

**Figure 8:**
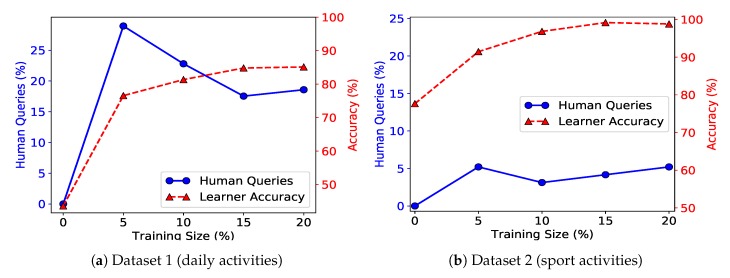
Total number of queries (active training data) versus number of human queries (left axis) and activity recognition accuracy (right axix). Subfigure (**a**) shows results for the daily activities dataset, and subfigure (**b**) shows results for the sport activities dataset.

The cost of active learning varies depending on the type of experts. In this subsection, we consider another real-world case where there are multiple on-demand experts in the system. As mentioned in Section 6.3, an on-demand expert provides label data with little or zero cost in the active learning phase. Each on-demand expert is trained in such a way that it has knowledge in one fourth of $\ell_{i}\in L$. Figure 8 shows the accuracy of the system for the case that we have an expensive expert and several on-demand experts (4 and 6 for daily and sport datasets). As we can see, as we move forward in the active learning process, the number of expensive queries remains below 15% and 5% for daily and sport activities datasets, while the accuracy increases steadily.

**Figure 9:**
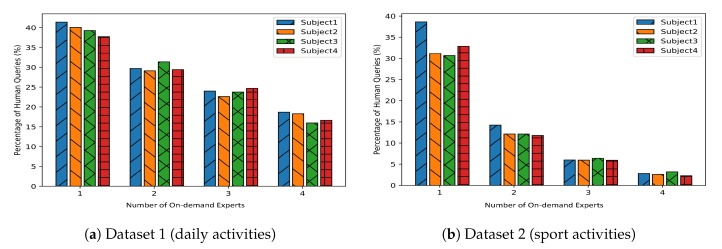
Number of on-demand experts versus number of queries from human expert(s).

Figure 9 shows how number of on-demand experts changes the number of queries from expensive experts. The higher the number of on-demand experts, the lower the number of queries. Figure 9a,b show the number of queries from the human for different number of on-demand experts. Higher number of on-demand experts covers a larger part of the data distribution, and consequently the number of queries from the human expert(s) decreases. The total number of queries is 10% for both datasets. The results are shown for different subjects.

#### 8.2.3. Benefits of Collaborative Active Learning

**Figure 10:**
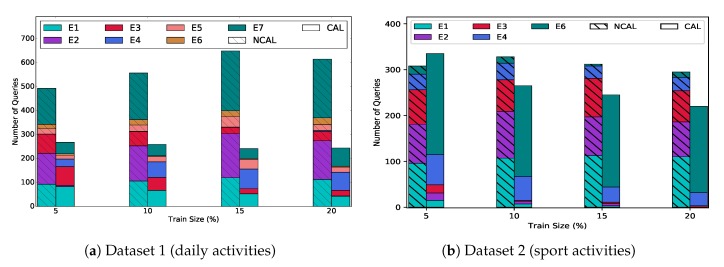
Comparison of non-collaborative and collaborative active learning in terms of total number queries. Each color corresponds to a different expert. The number of queries from imperfect (E2, E3) and perfect (E1) experts decreases in the collaborative learning because on-demand experts ((**a**) E4-E7, (**b**) E4 & E6) increase their knowledge in the previous rounds.

We simulate a real world scenario with all three types of experts. We set the cost for each expert proportional to their confidence on the label set *L*. The set of experts is sorted in a descending order based on their cost and we named them based on this order (E1 has the highest cost). Figure 10 presents the total number of queries for both collaborative (CAL) and non-collaborative (NCAL) algorithms. The goal of this experiment is to show how the collaboration between expert set reduces the cost, and increase the activity recognition accuracy.

The training data size corresponds to the number of queries from the set of experts. As we query more data instances from the set of experts, the queries from a human expert for the CAL algorithm decreases at a faster rate compared to the non-collaborative version (i.e., NCAL). Furthermore, confidence of on-demand and imperfect experts increases in CAL and the number of queries from them increase, and consequently the cost of data annotation is reduced.

## 9. Conclusions

We explored the use of active learning and transfer learning for reconfiguration of mobile health monitoring systems. Firstly, we proposed an expert selection algorithm which can be used in different real-world scenarios with heterogeneous set of experts. The expert selection module is incorporated as a core part of the expert management unit within the Co-MEAL architecture. The expert management unit cooperates with the query strategy module and the transfer learning (i.e., learner initialization) module to further decrease the cost of the active learning process. Secondly, the Co-MEAL architecture is designed to allow collaboration among experts in order to reduce annotation cost and enhance knowledge of inexpensive experts for future data labeling.

We demonstrated the efficacy of the expert selection algorithm within the Co-MEAL architecture using activity recognition as a case study. We designed several real-world scenarios for two different human activity datasets for cases where a system faces configuration change, context change or user need change. We showed that high accuracy (over 90%) can be attained by annotating only a small fraction (about 10%) of unlabeled data. We also showed that cost-optimal expert selection and collaboration among experts enables big savings in annotation cost.

In future work, we plan to extend the architecture to handle multi-modality sensory systems. We also plan to use the system in a real pilot study of an intervention system for diabetes patients. Our goal is to monitor their daily activities and collect daily and weekly questionnaires from patients using the proposed architecture implemented in a mobile phone app.
